# An Energy-Efficient and Robust Multipath Routing Protocol for Cognitive Radio Ad Hoc Networks

**DOI:** 10.3390/s17092027

**Published:** 2017-09-04

**Authors:** Kishor Singh, Sangman Moh

**Affiliations:** Department of Computer Engineering, Chosun University, 309 Pilmun-daero, Dong-gu, Gwangju 61452, Korea; kishorsingh@chosun.kr

**Keywords:** cognitive radio ad hoc network, routing, energy efficiency, stability, multipath, routing metric

## Abstract

Routing in cognitive radio ad hoc networks (CRAHNs) is a daunting task owing to dynamic topology, intermittent connectivity, spectrum heterogeneity, and energy constraints. Other prominent aspects such as channel stability, path reliability, and route discovery frequency should also be exploited. Several routing protocols have been proposed for CRAHNs in the literature. By stressing on one of the aspects more than any other, however, they do not satisfy all requirements of throughput, energy efficiency, and robustness. In this paper, we propose an energy-efficient and robust multipath routing (ERMR) protocol for CRAHNs by considering all prominent aspects including residual energy and channel stability in design. Even when the current routing path fails, the alternative routing path is immediately utilized. In establishing primary and alternative routing paths, both residual energy and channel stability are exploited simultaneously. Our simulation study shows that the proposed ERMR outperforms the conventional protocol in terms of network throughput, packet delivery ratio, energy consumption, and end-to-end delay.

## 1. Introduction

The unprecedented growth and development in the sphere of wireless communication and relevant applications have not only made spectrum a precious resource but also have severely undercut the concept of fixed spectrum assignment. The unlicensed bands, on one hand, are unexpectedly congested, whereas a large portion of the licensed bands are severely underutilized. The licensed spectrum bands such as ultrahigh frequency (UHF) and very high frequency (VHF) bands are not fully utilized, but, conversely, the unlicensed industry, scientific, and medical (ISM) bands are massively populated because of free access. This alarming situation has led to the birth of dynamic spectrum access [1], which aims at making the best opportunistic use of the unused spectrum called spectrum holes and thus ensures that both licensed and unlicensed bands are evenly utilized.

Cognitive radio technology has emerged as a promising and reliable remedy for improving spectrum utilization and resolving the spectrum problems. It has been exceedingly successful in realizing this urgency of wireless communication and leveraging the features of cognitive capability and reconfigurability [2]. The cognitive capability allows the cognitive radio to sense the surrounding radio environment and fetch information such as frequency, bandwidth, power, modulation technique, and communication technology, whereas cognitive reconfigurability enables the cognitive radio to redefine its internal state and parameters based on interactions with the outside world and thus adapt to the observed environment. As a result, cognitive radio technology allows cognitive devices to ensure opportunistic access to the portions of the unused spectrum band and adapt accordingly to achieve flawless communication flow.

A cognitive radio network (CRN) is basically populated by two types of users: licensed primary users (PUs) and cognitive secondary users (SUs). The PUs have prioritized possession over the licensed bands, whereas the SUs can use only the vacant channels unused by PUs and, more importantly, without interfering with the ongoing communication of PUs [3]. One of the most attention-seeking issues in CRNs, therefore, the uncertainty of spectrum availability resulting from PU activity and the subsequent challenge of accomplishing SU–SU communication without impeding PU transmission and optimal spectrum usage.

Cognitive radio ad hoc networks (CRAHNs) are an infrastructure-free form of CRNs. In fact, they are the enhanced form of conventional ad hoc networks, which are embedded with cognitive radio technology to ensure efficient and innocuous spectrum utilization [4]. The absence of a centralized network entity poses a number of challenges among them compared to their infrastructured counterparts. Without the network infrastructure, SUs must cooperate and communicate among themselves in an ad hoc fashion to exchange network-related information such as network topology, spectrum opportunities, and the presence of PUs [5].

Most existing CRAHN architectures assume that all SUs are equipped with cognitive radios and sense the radio environment. This cognitive functionality requires not only time and effort but also a significant amount of energy [6,7]. Channel availability in CRAHNs is different from that in conventional multichannel multihop networks such that SUs have partially overlapping and nonoverlapping sets of available channels, and the channels vary with time and space [8]. Furthermore, the distinguishing features such as dynamic topology, self-configurability, scalability, limited energy, and multihop architecture represent inevitable hurdles in realizing CRAHNs. A great deal of research and relentless effort is needed to cope with this challenging environment.

Routing in CRAHNs has emerged as one of the most sought-after topics for researchers worldwide. Because CRAHNs borrow attributes from both CRNs and ad hoc networks, the routing protocols must satisfy the requirements of both networks [9]. The unpredictable mobility of nodes, spectrum variation in time and space, energy constraints, and PU activities add burdens to the network layer protocols. A routing process in CRAHNs, therefore, must necessarily involve spectrum awareness, quality route setup based on some form of routing metric, and a route maintenance procedure to combat route failures, if any [10]. In addition, the interest of PUs must be protected, which can be best achieved by minimizing the interference experience due to imperfect spectrum sensing and spectrum allocation at SUs [11]. Furthermore, the sudden appearance of PUs forces SUs to vacate the current channel in use, whereas the mobility of SUs leads to potential route failures.

Multipath routing in CRAHNs allows SUs to switch dynamically among multiple paths whenever the normal routing is obstructed by either the appearance of PUs into the current channel or the movement of the node itself into the region of the PU. Multipath routing provides a set of primary and alternate paths to cope with route failures. The dynamic switching from the primary path to the alternate path can significantly reduce the frequency of the route discovery process and contribute to energy-efficient and reliable routing.

An energy-efficient routing scheme is highly needed for CRAHNs powered by energy-constrained batteries. In such a routing scheme, channel switching and rerouting should be minimized. When any PU appears, SUs should vacate the current channel in use, so a route that survives for a long period must be discovered. As a result, many factors including the residual energy of each participating node, the energy consumption along the path, the stability of the channel selected, and the backup path to counteract the sudden appearance of PUs must be considered. Such considerations can aid in achieving energy efficiency in routing.

Even though there are many routing schemes in the literature, most of the routing approaches handle node selection, energy awareness, spectrum decision or multipath routing separately. In this paper, the combined form of residual energy and route stability is exploited as the integrated route selection metric to discover energy-efficient and robust multiple paths while keeping in record the spectrum heterogeneity and primary user activity. That is, we propose an energy-efficient and robust multipath routing (ERMR) protocol that promises not only to conserve valuable energy with balanced energy consumption throughout the network but also to improve the robustness of routing paths. The channel selection algorithm integrated with route formation sets up a strong route with a long lifetime in terms of residual mode energy and link stability, resulting in improved energy efficiency and robustness. According to our extensive simulation results, the proposed ERMR outperforms the conventional protocol in terms of network throughput, packet delivery ratio, energy consumption, and end-to-end delay.

The rest of this paper is organized as follows: In the following section, related works are reviewed in brief. Section 3 depicts the system model for CRAHNs used in our study. In Section 4, the proposed ERMR protocol is presented along with its routing processes, and a qualitative comparison of various routing protocols is also addressed. In Section 5, the performance of the proposed ERMR is evaluated via computer simulation and compared with the conventional scheme. Finally, the paper is concluded in Section 6.

In the paper, some symbolic notations are used as follows: *S_t_*(*n*) denotes the state of a PU *n* at time *t*. *P_on_* and *P_off_* denote the probability of a PU being in ON and OFF state, respectively. Likewise, *u* and *v* represent the periods for which a PU is expected to be in ON and OFF states, respectively. *E_res_* and *S* are indicators for residual energy measure and route stability measure, respectively. *E_init_*(*n*) is the initial energy of node *n*, *E_con_*(*n*) is the energy consumed by node *n*, and *E_res_*(*n*) denotes the remaining energy of node *n*.

## 2. Related Works

Several routing protocols have been proposed to formulate energy-efficient data routing paths in the literature. More recently, several attempts to introduce multipath routing protocols have been reported.

The ad hoc on-demand multipath distance vector (AOMDV) [12] routing protocol is the basis of all multipath routing protocols in mobile ad hoc networks. This multipath version is an extension of the AODV [13] routing protocol and succeeds in providing a loop-free and disjoint set of paths.

The low-latency and energy-based routing (L2ER) protocol for CRAHNs [14] considers energy and delay simultaneously to select the optimal path for data transmission. This on-demand protocol uses joint route and spectrum selection to combat heterogeneity but fails to consider channel availability probability and stability of the link.

The joint path and spectrum diversity-based routing protocol (E-D2CARP) [15] exploits a multipath and multichannel environment to circumvent PU-affected regions and alleviate the PU occupancy problem. Nevertheless, it does not guarantee energy-efficient paths because no energy metric is involved in route selection. The main objective here is to combat the PU occupancy as it comes. It uses the expected path delay (EPD) metric to execute path selection, which stresses the path with minimal packet loss and the lowest end-to-end delay.

In [16], Beltagy et al. proposed a multipath routing protocol with an objective of improving the reliability of transmission paths in CRNs. The “route closeness” metric has been introduced to select the paths that are not close to each other. The main goal of this routing design is to ensure that a PU could not interrupt all selected nonclose paths at the same time. However, the protocol fails to address the issue of spectrum diversity, which is a distinguishing characteristic of CRAHNs. The protocol, thus, is not appropriate to be applied in highly dynamic CRAHNs.

In [17], a multipath routing protocol for CRAHNs is proposed, which aims at discovering resilient multiple paths in a single route discovery phase to enhance bandwidth utilization. The protocol stresses only the robustness of the path and hence selects the most stable path from source to destination. There is no consideration to the energy aspect of cognitive routing. It exploits the features of the MAODV route discovery mechanism with necessary modifications to fit into a cognitive radio environment.

It is difficult for a single routing metric to succeed in covering all design aspects of routing in complex networks such as CRAHNs. For example, the simple inclusion of an energy routing metric cannot ensure the robustness of the selected path, whereas the introduction of multiple paths in the form of backup paths helps with mobility-induced route failures and, eventually, contributes to the robustness of the data transmission procedure. On the other hand, random selection of the channels simply adds to the woes of spectrum heterogeneity both spatially and temporally. Most existing routing protocols fail to combine the metric-based channel selection with the path selection. The selection of channels not only contributes to improving the performance of the entire system but also allows leveraging of the multichannel environment.

A good routing protocol is highly dependent upon a good channel selection scheme [18]. A number of channel selection schemes such as the lowest interference impact on adjacent channels [19], channel availability probability [20], and contention-aware selection among the SUs [21] have been thoroughly applied. In this regard, using the availability time of the channel as a selection criterion can help enhance the stability of the link and the path as a whole. The allocation of the channel with the highest availability duration not only ensures route stability but at the same time lowers the frequency of channel switching, which is much needed in multihop multichannel networks. Furthermore, because CRAHNs are battery powered and highly dynamic, energy efficiency and stability are the main factors to be considered in the selection of routing paths in CRAHNs.

A good sensing scheme is inevitable in CRNs to enable the technology itself. Several approaches have been put forward so far to improve the process of spectrum sensing. The cognitive radio sensitivity can be well improved by enhancing radio frequency technology for wideband processing, exploiting digital signal processing techniques for PU detection, and implementing cooperation schemes allowing SUs to share their spectrum information [22]. The impact of user cooperation has been further investigated in [23], taking the context of spectrum sensing through multiple-input multiple-output (MIMO) decision fusion. However, the benefits of orthogonal transmission seem to be more powerful in the case of non-cooperative protocols, and almost negligible in the case of cooperative schemes.

All of the aforementioned works handle energy awareness, channel selection or multipath routing separately. As a consequence, CRAHNs could not be exploited to the fullest, resulting in limited performance. To cope up with this situation, we propose a routing protocol by addressing energy constraint, spectrum heterogeneity and dynamic topology at the same time. Unlike other protocols, the proposed ERMR exploits the combination of the threshold energy value, the channel selection based on channel availability and, ultimately, the cost function of residual energy and route stability in order to discover energy-efficient and robust routing paths. Moreover, the provision of the backup path is always an added advantage provided by the protocol in CRAHNs to improve the reliability. The competitive performance advantages of the proposed ERMR will be quantitatively presented and discussed in Section 5.

## 3. System Model

We consider a battery-powered CRAHN with *N* PUs and *M* SUs. The number of licensed channels is assumed to be equal to the number of PUs such that each PU has a particular channel assigned to it. PUs hold undisputable license for specific spectrum bands and do not deviate from their assigned spectrum bands. SUs are expected to exchange their network-related control information through a dedicated common control channel. SUs are assumed to possess two separate radio transceivers for control packets and data packets, respectively. It is also expected that SUs can tune their radio transceivers to any vacant PU channels. The number of vacant PU channels varies from one SU to another and with the passage of time. Therefore, SUs are supposed to have the spectrum sensing capability to determine the vacant channels not in use by PUs and the ability to use them opportunistically. In this paper, we assume that PUs are static whereas SUs can be mobile. The SU mobility that concerns PUs is dealt with by constantly checking if the SU is within the transmission range of PUs. If a SU moves away from the PU in ON state such that it goes beyond transmission range, then the SU is able to use the corresponding channel. On the other hand, if a SU moves into the transmission range of the PU in ON state, then it drops the corresponding channel and switches to the next channel.

**Figure 1:**
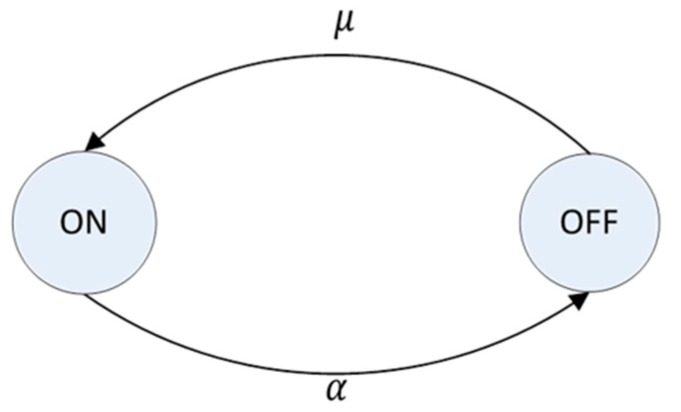
Alternating ON/OFF states of primary users (PUs).

Good PU activity modeling is important to make the environment more realistic. PUs are modeled by virtue of independent and identically distributed ON and OFF process with exponential distribution [24]. Figure 1 shows the alternating ON and OFF states of a PU. In ON state, the PU is active and occupies its channel for period $T_{ON}$, during which it performs its data transmission via that channel; thus, SUs are not allowed to use the channel. Conversely, in OFF state, the PU is inactive, so SUs can temporarily use the licensed channel until the PU becomes active. The state of PU *n* at time *t*, *S_t_*(*n*), is active (1) or inactive (0) and can be represented as

(1) $$S_{t}(n)=\{\begin{array}{ll} 1 & \mathrm{if}\;\mathrm{PU}\;\mathrm{is}\;\mathrm{active}\\ 0 & \mathrm{otherwise}. \end{array}$$

If the ON and OFF periods of a PU are exponentially distributed with mean ON period $\frac{1}{\alpha }$ and mean OFF period $\frac{1}{\mu }$, where *α* is the departure rate of the PU and *μ* is the arrival rate of the PU, then the probability of the PU being in ON state, *P_on_*, is given by

(2) $$P_{on}=\frac{\mu }{\mu +\alpha }$$

Obviously, the probability of the PU being in OFF state, *P_off_*, is 1 − *P_on_* and can be represented as

(3) $$P_{off}=\frac{\alpha }{\mu +\alpha }$$

On the other hand, *P_on_* and *P_off_* follow an exponential distribution. Given ON period *u*, therefore, the probability of the PU being in ON state, *P_on_*(*u*), is given by

(4) $$P_{on}(u)=1-e^{-\lambda u}$$

where *λ* is the mean ON period of the PU (i.e., *λ* = $\frac{1}{\alpha }$). Given OFF period *v*, the probability of the PU being in OFF state, *P_off_*(*v*), is given by

(5) $$P_{off}(v)=1-e^{-\gamma v}$$

where *γ* is the mean OFF period of the PU (i.e., *γ* = $\frac{1}{\mu }$). From Equation (4), the ON period *u* of a PU can be represented by

(6) $$u=\frac{-\ln(1-P_{on}(u))}{\lambda }$$

From the Equation (5), the OFF period *v* of a PU can be represented by

(7) $$v=\frac{-\ln(1-P_{off}(v))}{\gamma }$$

## 4. Energy-Efficient and Robust Multipath Routing

In this section, the proposed ERMR protocol for CRAHNs is presented in detail. In ERMR, energy-efficient primary and backup paths are built to counteract route failures for a source-destination pair. The route discovery process starts only when both primary and backup routes break down. The selection of next-hop nodes based on their residual energy not only conserves their energy for latter sessions but also ensures balanced energy consumption throughout the network. It is guaranteed that the paths considered are the most energy-efficient paths with respect to the energy consumed in transmission, reception, channel switching, and idle listening.

It is worth noting that the route discovery procedure is carried out in collaboration with the channel selection. At each link, the selection of the next-hop node is performed simultaneously with channel allocation. The assignment of a better channel to each link can boost the overall performance of the network. Hence, the channel assigned is the most stable one in terms of access duration. The proposed ERMR protocol consists of route discovery, route reply, data transmission, and route maintenance phases.

### 4.1. Route Discovery

The process of route discovery allows the source to find the multiple routes between itself and the destination based on the routing metric. In the proposed ERMR, route discovery is a daunting twofold task that considers the residual node energy and the spectrum availability. In principle, it is based on the AOMDV routing algorithm. However, for adapting a cognitive radio environment, there are some modifications.

**Figure 2:**
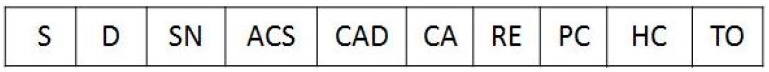
RREQ packet structure. S: Source; D: Destination; SN: Sequence Number; ACS: Available Channel Set; CAD: Channel Access Duration; CA: Channel Assigned; RE: Residual Energy; PC: Path Cost; HC: Hop Count; TO: Timeout.

When a node has data to transmit, it searches a valid routing path for the intended destination in its routing table. If no valid routing path is found, the node starts the route discovery process. In this process, a route request (RREQ) packet is broadcasted from the source to all its one-hop neighbors, forming multiple reverse paths at intermediate nodes. Every intermediate node that receives a RREQ packet checks if it is the intended destination; if not, it broadcasts the RREQ packet further. At the destination, a route reply (RREP) packet is unicasted towards the source along the reverse paths formed by RREQ forwarding. The multiple paths formed are loop-free and node-disjoint. That is, flood-based route discovery and regular route updates ensure loop freedom and path disjointedness. Figure 2 shows the basic structure of the RREQ packet.

**Figure 3:**
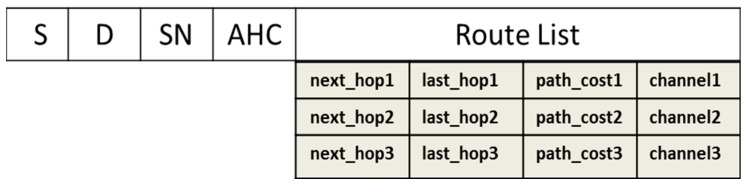
Routing table structure. S: Source; D: Destination; SN: Sequence Number; AHC: Advertised Hop Count.

Because the route discovery is a hop-by-hop process, the regular update of RREQ fields is mandatory. Along with this, there is a routing table to be maintained at each participating node from source to destination. The routing table should hold the information represented in Figure 3 to ease the route discovery and subsequent selection.

For every route discovery, the source broadcasts an RREQ packet via a common control channel (CCC). The RREQ packet exists only for the period defined by the TO field in Figure 2. When a neighboring node receives the RREQ packet, it first checks whether its battery energy exceeds the threshold value and whether there is any common vacant channel. Note that the communicating two nodes of transmitter and receiver not only should be within each other’s transmission range but also must possess at least one common vacant licensed channel between them. If the two conditions are not met, the packet is dropped; otherwise, the intermediate node responds accordingly.

In the response process, it first checks whether it is the destination being sought. If so, it replies with a unicast RREP packet; otherwise, it rebroadcasts the RREQ packet into its surroundings. Before it actually rebroadcasts the RREQ packet, the routing table is updated with information such as the last hop node, hop count for the reverse path, and channel assigned for that particular link. The channel assigned is the most stable channel available for that link. Likewise, the RREQ packet itself is also updated by appending node ID, available channel set, residual energy, most stable channel, path cost, and hop count (residual energy, channel stability, and hop count are increased by adding to their previous values). This process continues until the destination is reached, where the route decision and selection are made.

To enable multiple paths between source and destination, the concept of sequence number is exploited, which facilitates unique identification for an RREQ packet. To ensure that the routes are disjointed, the redundant RREQ packets with the same sequence number as an RREQ packet that the node has already forwarded should be ignored.

**Figure 4:**
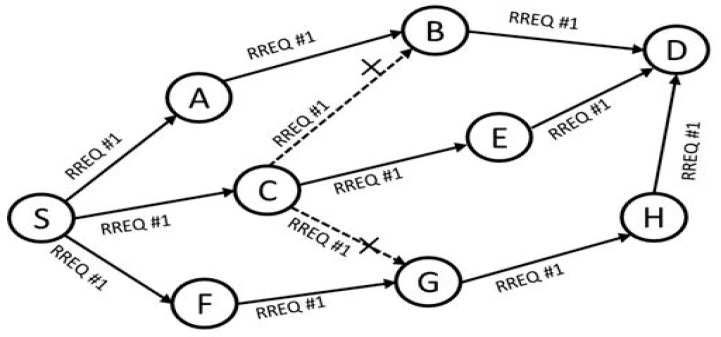
Multipath routing path discovery.

Figure 4 shows an example procedure of the route discovery process, where three disjoint routing paths can be found. As seen in the figure, the RREQ packet forwarding begins from source S and ends at destination D. Each of the nodes ensures that no RREQ packet is forwarded twice to avoid looping and maintain path disjointedness. In Figure 4, nodes B and G drop the RREQ packets with the same sequence number #1 as the RREQ packet that they have already forwarded. This helps maintain the concept of disjoint and loop-free paths, which is crucial in multipath routing. The destination responds to only the RREQs received within the time set in the timer at the destination. All RREQs received after the time specified are dropped automatically.

### 4.2. Route Decision

At the destination, when RREQ packets are received from multiple paths, they must be sorted according to their energy efficiency and stability. This helps categorize the paths as primary and backup paths. We use a cost function, a combination of energy and stability metrics, to evaluate the paths. The cost function has no dimension (even though the residual energy is measured in Joules and the route stability is measured in seconds). That is, as for the evaluation of candidate paths, we have taken into account only the numeric values of residual energy and route stability and used them in the cost function. For both residual energy and route stability, the higher is the better. Hence, they are summed up. The path with the highest cost function is chosen as the primary path, and that with the second-highest cost function is used as the backup path. The cost function is as follows:

(8) $$\mathrm{Cost}\;\mathrm{Function}(\mathrm{CF})=a\cdot E_{res}+b\cdot S,$$

where *E_res_* and *S* are the residual energy and stability of the path, respectively, and *a* and *b* are *α*/*E_init_* and *β*/*S*_max_, respectively, with the condition of *α* + *β* = 1. The two constants *α* and *β* are the weight factors of residual energy and stability, respectively. It should be noticed that *E_init_* is the sum of the initial energy of nodes in the path (i.e., $E_{init}=\sum_{n=1}^{m}E_{init}(n)$), where *E_init_*(*n*) is the initial energy of node *n* in the path of *m* nodes, and *S*_max_ is the sum of the average ON/OFF period of links in the path (i.e., $S_{\max}=\sum_{l=1}^{k}T_{on}(l)+T_{off}(l)$), where *T_on_*(*l*) + *T_off_*(*l*) is the average ON/OFF period of link *l* in the *k*-hop path. The values of *α* and *β* are assigned according to the significance of residual energy and stability in the target application. In our performance simulation in Section 5, *α* = 0.5 and *β* = 0.5 by default.

The residual energy of the path refers to the sum of the residual energy for every node on the path, and it is given by

(9) $$E_{res}=\sum_{n=1}^{m}E_{res}(n),$$

where *E_res_*(*n*) is the residual energy of node *n,* and *m* is the number of nodes in the path including the source and the destination. In order to avoid the selection of severely energy-deprived nodes, we set up a threshold energy level such that the nodes with energy level below threshold are not included in the route during route discovery process. This strategy is intended to achieve an even load distribution over the network. *E_res_*(*n*) is the difference of the initial energy of node *n* (*E_init_*(*n*)) and the total energy consumed by node *n* (*E_con_*(*n*)) as indicated below:

(10) $$E_{res}(n)=E_{init}(n)-E_{con}(n),$$

The transmission of a packet from one node to the next relay node is an energy-consuming process. Energy is consumed not only at the transmission but also at the receiving side. If the transmission and reception occur through different channels, a considerable amount of energy is involved in the switching process. The energy incurred in channel switching is proportional to the frequency difference between the channels [25]. Likewise, a dominant proportion of radio energy is consumed when the radio is listening to the channel in order to receive possible data. Often, idle listening cost is speculated to be 50–100% of the energy required for the receiving purpose. Therefore, we consider all aforementioned factors to gauge the energy. If we neglect overhearing and assume data transmission to be completely along the path specified, the energy consumed by a node, *E_con_*, can be calculated by

(11) $$E_{con}=E_{tx}+E_{rx}+E_{swt}+E_{idle}.$$

where *E_tx_*, *E_rx_*, *E_swt_*, and *E_idle_* are represented as follows:

- *E_tx_* is the energy required for transmitting a packet and is given by $E_{tx}$ = (1.65 × packet size in bits)/(2 × 10^6^) J.
- *E_rx_* is the energy required for receiving a packet and is given by $E_{rx}$ = (1.15 × packet size in bits)/(2 × 10^6^) J.
- *E_swt_* is the energy consumed for channel switching and is given by *E_swt_* = *P_sw_* × *t_sw_* × |*F*_2_ − *F*_1_|, where *P_sw_* is the power dissipation for channel switching, *t_sw_* is the time for channel switching of the unit bandwidth, and *F*_2_ and *F*_1_ are the frequencies of the channels switched to or from, respectively.
- *E_idle_* is the energy loss while listening to a channel and is given by *E_idle_* = 0.8 × *E_rx_*.

Regarding the stability of the path, we consider the time during which PU is in OFF state for the next time period such that the corresponding channel can be used by SUs. In other words, it is the access duration available for SU communication. The higher the access duration, the more stable the link. Hence, the stability, *S*, can be measured as

(12) $$S=\sum_{l=1}^{k}T_{off}(l),$$

where *k* is the number of links in the path, and *T_off_*(*l*) is the period during which a channel is available to SUs for link *l*, which follows from Equation (7).

We use this approach to evaluate the cost function for each path. Consequently, the path with the maximum value of the cost function is taken as the primary path. The backup path is the path with the second-highest value of the cost function, and it is taken in case the primary path suffers from route failure.

### 4.3. Route Reply

**Figure 5:**
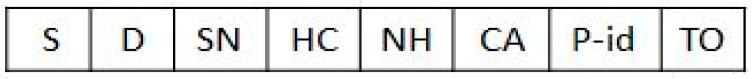
RREP packet. S: Source; D: Destination; SN: Sequence Number; HC: Hop Count; NH: Next Hop; CA: Channel Assigned; P-id: Path identifier; TO: Timeout.

The selected primary and backup paths are sent back to the source by the destination via the route reply process. The destination node, upon receiving the RREQ copies, forms the reverse paths similar to the process at intermediate nodes. It unicasts separate route reply (RREP) packets to each of the primary and backup paths discovered. The basic structure of the RREP packet is depicted in Figure 5. The main field that should be focused on in the RREP packet are P-id (path identifier), CA (channel assigned), and NH (next hop), which indicate the preference order of the selected paths, the channel identifier, and the next hop node identifier for the forward path, respectively.

When an intermediate node receives an RREP packet, it sets up the forward path along the channel selected and then forwards a copy of the RREP packet along the reverse path stored in its routing table; at the same time, it updates the hop count, the channel assigned, and the next hop field of the route reply packet. The routing table of intermediate nodes is also updated with the information in the RREP packet to set up the forward path. If an intermediate SU node receives a duplicate of the RREP packet for the same source–destination pair, the packet is dropped except when it is for the new route discovery session.

### 4.4. Route Maintenance

Route failures in CRAHNs occur primarily because of two events: one is the sudden arrival of a PU into the channel being used by the SU pair, and the other is the breakage of formed routes due to node mobility. The two failures are quite different with respect to the source of failure origins. Therefore, the routing protocol must be sufficient to determine the cause and provide an appropriate solution. If the channel being used is restored by the corresponding PU, then the data transmission on the channel is subjected to halt immediately. A new channel is explored to continue the transmission of SUs as mentioned in the channel selection procedure. If the route failure occurs owing to the dynamic behavior of any mobile node, the routing path must be changed. The data routing should shift to the backup path. Failure notification of any route during the process should be sent to all participating nodes via some information flow, which can be in the form of HELLO packets.

### 4.5. Qualitative Comparision of Routing Protocols

A qualitative comparison of different routing protocols is presented in Table 1 with respect to different aspects.

Each protocol uses a different routing metric to select the best routing path in terms of the metric. AOMDV is a shortest-path routing protocol that uses hop count as a routing metric. L2ER, on the other hand, has a cumulative routing metric that tends to select the optimal path with the minimal energy consumption and end-to-end delay combined. E-D2CARP stresses the probability of packet loss and link delay in its routing procedure via its routing metric called expected path delay (EPD). PMRC ensures that the selected routes are not close enough to be interrupted by an active mobile PU. Route closeness is the measure for the closeness of the routing paths in this protocol. MRPC based on MAODV uses hop count to select the shortest routes, whereas channel stability ensures that the channel assigned is the most stable one. The proposed ERMR uses a hybrid metric, a combination of energy consumption and channel stability, to evaluate routing paths and channel assignment. The path with the maximal residual energy is taken as the most preferred routing path. For a link, the channel that ensures the highest stability is chosen.

AOMDV uses a single channel for both control message and data dissemination. L2ER considers the channels that are less interfered from PU communication. E-D2CARP tends to make use of the channels currently not occupied by any PU. The channel assigned to a particular link is the one from which the control packet is received. The same channels are used for control packet exchange among SU nodes when available. In PMRC, there is a channel for each of the data and control messages. The proposed ERMR selects the most stable channel from the set of available channels for data transmission.

Regarding energy, L2ER and the proposed ERMR aim at improving energy efficiency. In the proposed ERMR, multiple paths are explored between source and destination. The nodes are examined for their residual energy level while the channels are formed for their free access duration. Eventually, the path with the best routing metrics designed is set up as the primary routing path. The backup or alternate path is the one with the second-best routing metrics.

## 5. Performance Evaluation

In this section, the proposed ERMR is evaluated via extensive computer simulation using NS-2.31 [26]. ERMR is compared to the conventional L2ER because L2ER is also an energy-efficient routing protocol for CRAHNs and exploits multiple routing metrics as in the proposed ERMR. In principle, L2ER tends to find the best path by avoiding the tradeoff situation between the path with high residual energy but significant end-to-end delay and the path with minimal end-to-end delay but low residual energy. In the following subsections, the simulation environment, performance metrics, and simulation results are presented and discussed.

### 5.1. Simulation Environment

We consider a network area of 1000 × 1000 m^2^ where a set of PUs and SUs are randomly deployed. In addition, we consider 11 channels, one of which is dedicated as a control channel and the rest are assigned to PUs as licensed channels. The PU activity is based on an ON/OFF model in which the ON and OFF times of PUs are exponentially distributed. During the ON state, the PU occupies the channel assigned for the calculated time and, conversely, frees the same channel during the OFF state. The transmission ranges of PUs are set to 150 m and are assumed to be completely static. On the other hand, SUs have the transmission range of 250 m and are expected to move in a random direction with a random speed within a range of 0–5 m/s and a pause time of 30 s. All routing control packets are transmitted through a dedicated CCC, which is shared by all SUs. The data transmission is accomplished through the data channel selected from the channel selection policy. The weight factors of residual energy and stability are set as *α* = 0.5 and *β* = 0.5. The rest of the simulation parameters are listed in Table 2.

### 5.2. Performance Metrics

The following performance metrics are evaluated to measure the performance of the proposed routing protocol.

- Average network throughput: This refers to the total number of successfully received bits per second over the number of source-destination pairs.
- Packet delivery ratio (PDR): This is the ratio for the number of successfully delivered packets to the destinations over the number of packets sent from the sources. It can be regarded as a metric for routing path reliability.
- Average energy consumption per bit: This indicates the average amount of energy in joules consumed for a bit of a data packet to reach the destination successfully.
- Average end-to-end delay: This is the average time a packet takes to reach its destination once generated from the source.

### 5.3. Simulation Results and Discussion

In this subsection, the simulation results of ERMR and L2ER are comparatively discussed. By varying the number of active sessions and the number of SUs, the abovementioned four performance metrics are evaluated and compared.

#### 5.3.1. Varying the Number of Sessions

The number of active sessions is increased from 4 to 16 with a step size of 2, and its impact on the network throughput, PDR, average energy consumption per bit, and average end-to-end delay are observed and analyzed. The SUs are subjected to a random velocity between 0 and 5 m/s, and the pause time is set to 30 s, whereas the number of SUs is fixed to 30. That is, we evaluate the impact of different traffic scenarios on the performance of the routing protocols.

**Figure 6:**
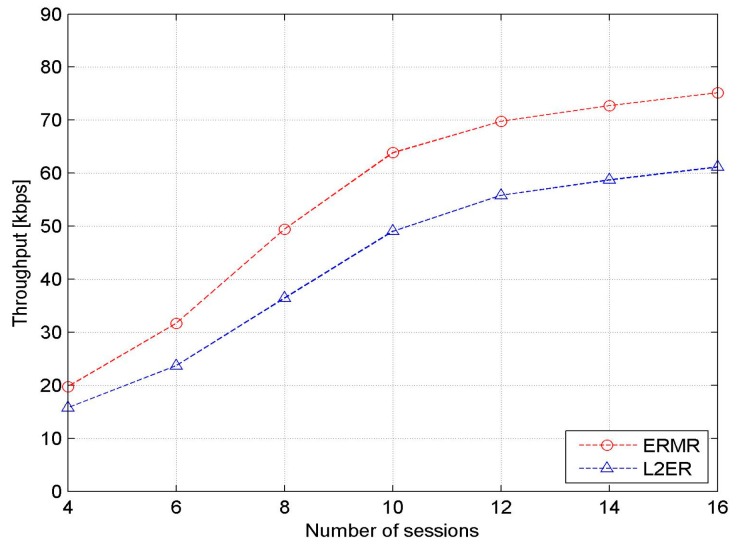
Network throughput vs. the number of sessions.

Figure 6 shows the average network throughput as a function considering the number of active sessions. As shown in the figure, the throughput of both protocols is enhanced with increasing number of sessions. This is because the greater the number of source–destination pairs, the greater the number of packets successfully delivered to their destinations within the specified time. In addition, with a smaller number of connections, the throughput difference between ERMR and L2ER is small. Nevertheless, the proposed ERMR outperforms L2ER significantly with the greater number of sessions. ERMR has up to 25% higher throughput than L2ER. The proposed protocol with multiple paths has a better tendency to combat the dynamic environment and the stress of heavy traffic.

**Figure 7:**
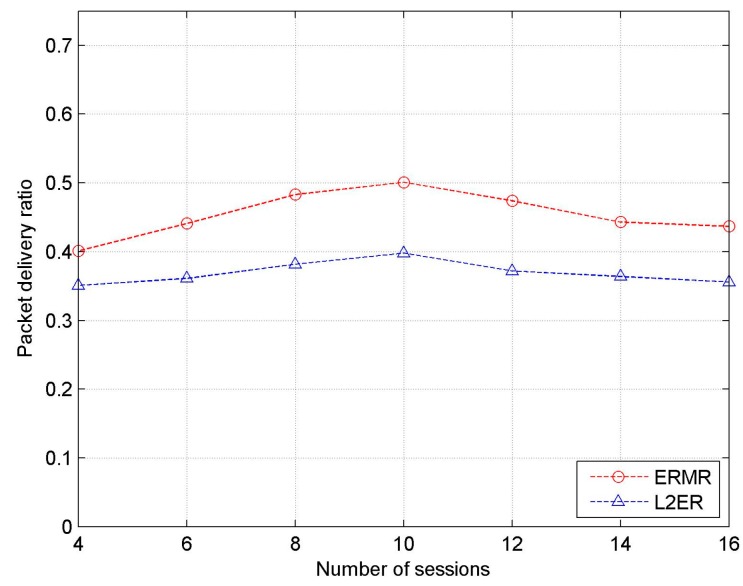
Packet delivery ratio vs. the number of sessions.

Figure 7 shows the PDR as a function considering the number of active sessions. It should be noted that the increases in the number of sessions has dual effects on PDR. That is, PDR increases with increasing number of active sessions, and it reaches its peak value at 10 active sessions. Thereafter, PDR decreases owing to the traffic congestion in the network. The proposed ERMR depicts better PDR by a healthy margin of 20–25% in all cases, proving its robustness in CRAHNs. The provision of backup paths for responding to route failures and the availability of more stable channels contribute to the improvement in the data delivery percentage.

**Figure 8:**
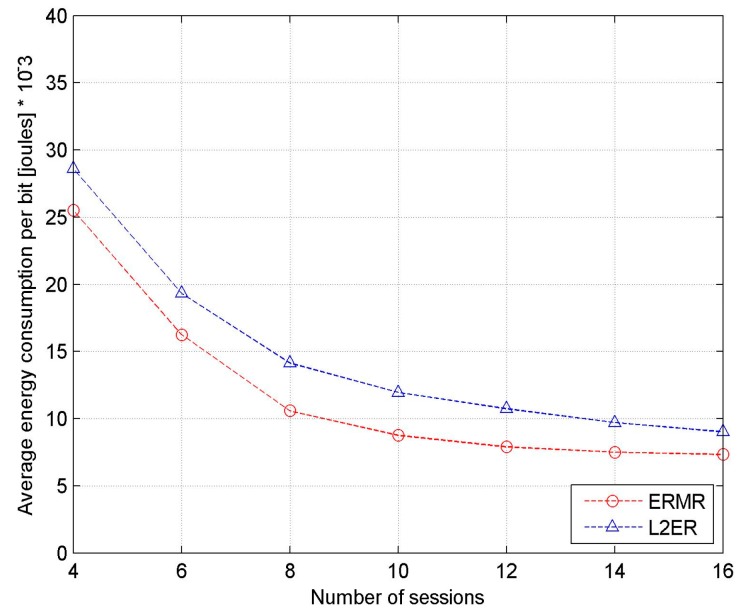
Energy consumption per bit vs. the number of sessions.

Figure 8 shows the average energy consumed per bit as a function considering the number of active sessions. It is found that the energy consumption per bit is sharply decreased until the number of sessions is 10 and thereafter attains almost steady consumption. In fact, the amount at which average energy consumed per SU increases with additional sessions is considerably low compared to the number of packets reaching the destinations. This is what leads to the trend of the graph in the figure. Furthermore, we find that the proposed routing protocols exhibit uniformly low energy consumption throughout the process. Basically, both routing protocols are energy-efficient routing protocols designed for CRAHNs. However, L2ER does not consider the channel switching energy, which is inevitable in multichannel networks. In addition, a significant amount of energy can be saved for each route discovery process that takes place in mobile networks. Consequently, using the backup path preserves the improvident energy consumption in discovering a new routing path at every route failure. Likewise, the channel selection policy in the proposed ERMR avoids frequent channel switching by ensuring a stable channel for data communication, resulting in improved energy conservation.

**Figure 9:**
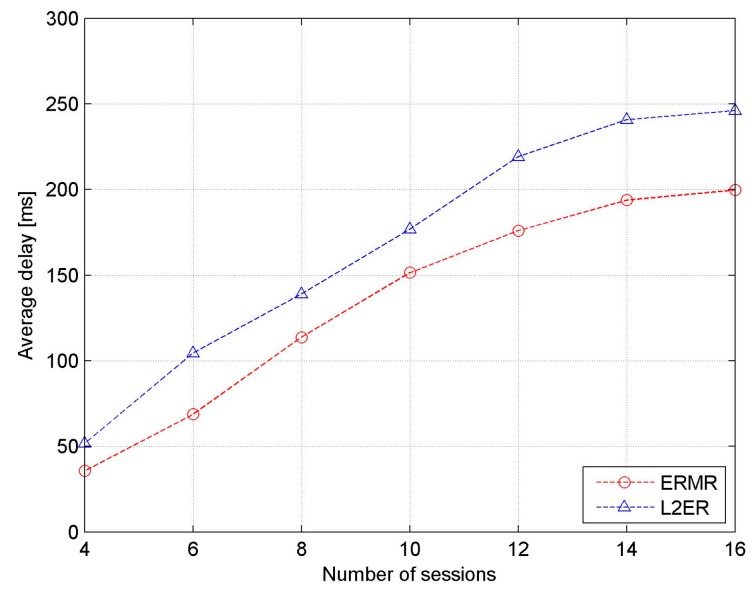
Average end-to-end delay vs. the number of sessions.

Figure 9 shows the average end-to-end delay as a function considering the number of active sessions. For both protocols, the average end-to-end delay increases almost linearly with increasing number of sessions. It is also obvious that the end-to-end delay of the proposed ERMR is lower than that of L2ER. This results from the better adjustment to the dynamic network and spectrum heterogeneity in ERMR.

#### 5.3.2. Varying the Number of SUs

We vary the number of SUs from 10 to 45 with a step size of 5 in a fixed network size and study its impact on the average network throughput, PDR, average energy consumption per bit, and average end-to-delay. The number of active sessions in each case is fixed at 10, making SUs mobile with speed randomly chosen between 0 and 5 m/s.

**Figure 10:**
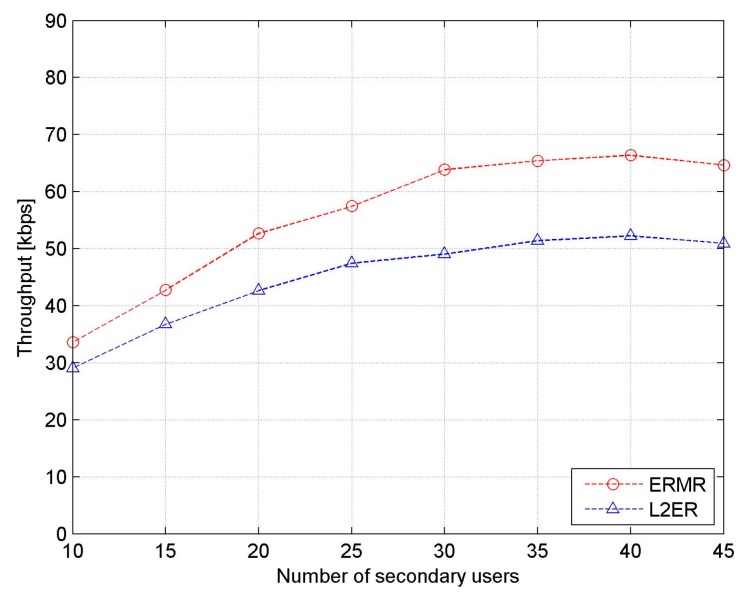
Network throughput vs. the number of secondary users (SUs).

As shown in Figure 10, it is observed that the average throughput of both protocols follows the same trend with increasing number of SUs. The throughput increases quite noticeably in the first half, resulting in the almost steady state in the second half. As the number of SUs increases, the throughput of ERMR is increasingly better than that of L2ER. That is, at low SU density, the proposed ERMR outperforms the existing L2ER protocol by approximately 15% and, at high SU density, the gap becomes approximately 30%. This allows us to infer that ERMR has better adaptability and robustness to the increase in the node density of SUs. This is mainly because the proposed ERMR with multiple paths effectively negates the impact of possible route breakages in mobile networks, and the number of channel switching events is significantly reduced in the multichannel environment.

**Figure 11:**
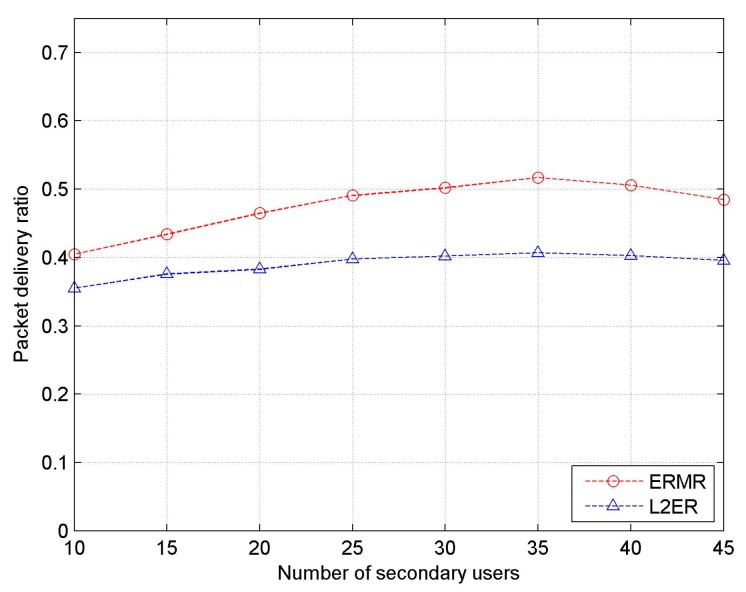
Packet delivery ratio vs. the number of SUs.

Figure 11 depicts PDR as a function of SU density. With increasing number of SUs, the trend of PDR is very similar to that of throughput shown in Figure 10. That is, the plots gradually increase in the beginning, are seemingly steady in the middle, and slightly drop in the end. As in the case of throughput, the gap in the two PDR lines is low at low SU density and tends to widen with increased SU density because of better resilience to mobility effects. The lower the route failure, the greater the number of packets reaching the destination. In addition, the longer spectral duration also contributes to improving the successful packet transmissions because it provides healthy routes for a longer period.

**Figure 12:**
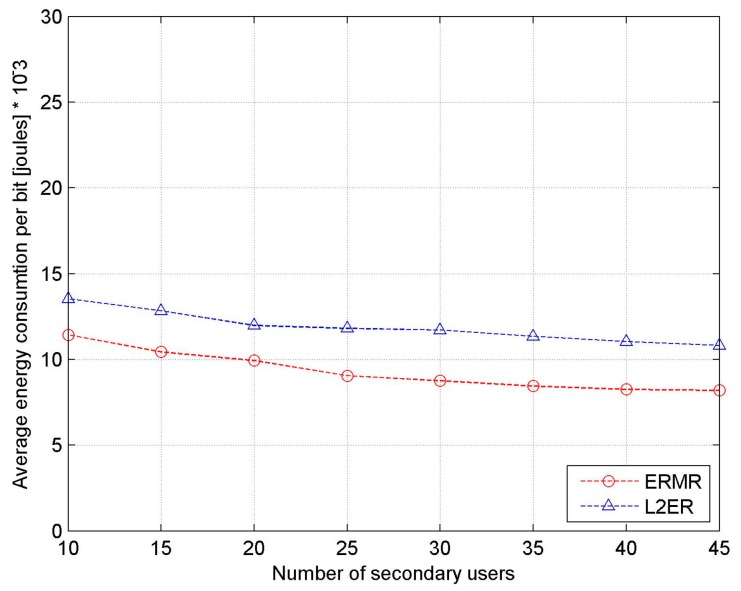
Energy consumption per bit vs. the number of SUs.

Figure 12 shows the energy consumption per bit with the variation in the number of SUs. It shows a gradual decline in the energy consumption per bit for both ERMR and L2ER. This is because, although the average energy consumed per node is increased, the number of successfully delivered data packets is increased even further. That is, the increasing rate of data delivery is much better than that of energy consumption. Clearly, the proposed ERMR protocol proves its advantage in saving much energy owing to its reduced route discovery processes, robustness, and reduced channel switching activities.

**Figure 13:**
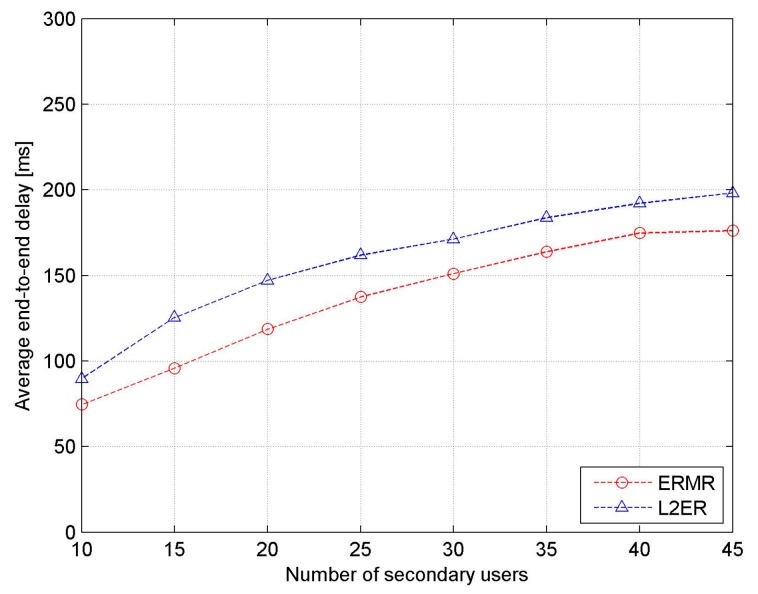
Average end-to-end delay vs. the number of SUs.

It is shown in Figure 13 that, with increasing number of SUs, the average end-to-end delay suffers a rapid increase in both protocols. Thanks to the better adaptability to the mobile network scenarios, however, the proposed ERMR has lower latency, fewer link failures, lower delays involved in the data transmission process, and less switching delay in the entire routing process.

## 6. Conclusions

In this paper, we have proposed an energy-efficient and reliable multipath routing protocol named ERMR for CRAHNs. Owing to the dynamic nature and unexpected PU behavior in CRAHNs, both energy efficiency and reliability are primarily emphasized, and the well-known performance metrics of throughput and delay are primarily considered in designing ERMR. As a result, the proposed ERMR protocol is on-demand, energy-efficient, robust, and reliable with multipath routing capability. According to our simulation results, the proposed ERMR outperforms the conventional protocol significantly in terms of network throughput, packet delivery ratio, average energy consumption per bit, and average end-to-end delay. In the simulation environment of our performance study, PUs are assumed to be static. As a future work, we plan to consider the mobility of PUs. Also, we are going to exploit other possible metrics in the performance simulation in the near future.

## Figures and Tables

**Table 1:** Qualitative comparison of routing protocols for cognitive radio ad hoc networks (CRAHNs).

Protocol	Routing Metric	Channel Selection	Energy	CCC	Advantage	Limitation
AOMDV [12]	Hop count	Single channel	No	No	Ability to cope with mobility route failures	Less effective in low-density networks
L2ER [14]	Energy and delay (cumulative)	Interference measure	Yes	Nondedicated	Appropriate for energy- and delay-aware applications	Poor performance in mobile environments
E-D2CARP [15]	Expected path delay	PU unoccupied	No	Nondedicated	Less vulnerable to PU activities	More control overhead
PMRC [16]	Route closeness	Single data channel	No	Dedicated	High link reliability	No multichannel support
MRPC [17]	Hop count and stability	Stability	No	Dedicated	Resilient to spectral heterogeneity	No energy awareness
ERMR	Energy and stability	Channel availability time	Yes	Dedicated	Suited for mobile networks	Static (nonmobile) PUs

**Table 2:** Simulation parameters.

Parameters	Value
Simulator	NS-2.31
Network area	1000 × 1000 m^2^
Number of primary users	10
Number of secondary users	10, 15, 20, 25, 30 (default), 35, 40, 45
Number of licensed channels	10
PU modeling	ON/OFF model
Traffic type	CBR
Packet size	512 bytes
Mobility	Random waypoint
Speed	0–5 m/s
Pause time	30 s
Number of sessions	4, 6, 8, 10 (default), 12, 14, 16
Initial energy	Randomly chosed (750–760 J)
Transmission power	1.15 W
Reception power	1.0 W
Ideal power	0.8 W
Switching power	0.8 W
Simulation time	250 s
